# Late recurrence of pStage 1 low-grade serous ovarian tumor presenting as a symptomatic bone metastasis: a case report

**DOI:** 10.1186/s13000-018-0720-1

**Published:** 2018-06-30

**Authors:** Chiaki Kubo, Shigenori Nagata, Takeshi Fukuda, Rieko Kano, Takaaki Tanaka, Katsuyuki Nakanishi, Masahiko Ohsawa, Shin-ichi Nakatsuka

**Affiliations:** 1 Department of Diagnostic Pathology and Cytology, Osaka International Cancer Institute Hospital, 3-1-69 Otemae, Chuo-ku, Osaka, 541-8567 Japan; 2 Department of Obstetrics and Gynecology, Osaka City University Graduate School of Medicine, Osaka, Japan; 3 Department of Orthopedic Surgery, Osaka International Cancer Institute Hospital, Osaka, Japan; 4 Department of Diagnostic and Interventional Radiology, Osaka International Cancer Institute Hospital, Osaka, Japan; 5 Department of Diagnostic Pathology, Osaka City University Graduate School of Medicine, Osaka, Japan

**Keywords:** Ovarian cancer, Serous borderline tumor, Atypical proliferative serous tumor, Low-grade serous carcinoma, Late recurrence, Bone metastasis, BRAF, KRAS

## Abstract

**Background:**

Ovarian serous borderline tumor/atypical proliferative serous tumor (SBT/APST) is characterized by presenting at an early stage and much longer survival than high-grade serous carcinoma. Given that the prognosis of ovarian SBT/APST with no invasive features is excellent, remote relapse after surgery can pose a diagnostic pitfall. Bone metastasis as transformed low-grade carcinoma is an extremely rare initial presentation of recurrence in patients whose primary tumor was confined to the ovaries.

**Case presentation:**

A 55-year-old Japanese woman who had undergone surgery for a right ovarian tumor 13 years previously presented with right-lateral chest pain and neurologic abnormalities in the lower limbs. Computed tomography (CT) scan and magnetic resonance imaging revealed an irregular mass in the right arch of the 12th thoracic vertebra, extending through the intervertebral foramen and into surrounding soft tissue, the maximum diameter of the whole mass being 78 mm. Pathological examination of a CT-guided needle biopsy of the paraspinal lesion demonstrated papillary cell clusters with blunt nuclear atypia and psammomatous calcification that were positive for PAX8, estrogen receptor, and WT1, but negative for thyroglobulin on immunohistochemical testing, and of a P53 non-mutational pattern. On clinicopathologic review, the previous 13- × 11- × 9-cm ovarian tumor was an intracystic and exophytic papillary growth without surface involvement; it had ruptured intraoperatively. Microscopically there was serous epithelium with minimal cytologic atypia proliferating in hierarchical branches with no invasive foci or micropapillary components. The tumor was confined to the right ovary with no peritoneal implants. Neither primary nor metastatic tumor harbored KRAS/BRAF mutations according to polymerase chain reaction using formalin-fixed paraffin-embedded tissues. We concluded that, after a 13-year disease-free interval, the paraspinal lesion was bone metastasis of low-grade carcinoma originating from the ovarian SBT/APST. The patient received radiotherapy for the paraspinal lesion followed by administration of paclitaxel and carboplatin plus bevacizumab and remains alive 168 months after the initial surgery.

**Conclusions:**

Pathologists and radiologists should not exclude late recurrence of ovarian SBT/APST when bone metastases are suspected, even when neither peritoneal nor lymph node involvement are detected. Long-term surveillance of women with ovarian serous tumors with no invasive features is recommended.

## Background

Serous borderline tumor/atypical proliferative serous tumor (SBT/APST) comprises approximately 50% of ovarian borderline tumors and is characterized by young age at diagnosis, slow growth, and progression to relatively indolent low-grade serous carcinoma (LGSC) [1]. Typically, the prognosis of ovarian SBT/APST with no invasive features is excellent in patients undergoing optimal reduction surgery; nevertheless, its predilection for remote recurrence poses a major diagnostic pitfall. We report a patient with ovarian SBT/APST found to have transformed into LGSC as a metastasis to the thoracic vertebra with infiltration of surrounding soft tissue 13 years after removal of the primary tumor confined to the right ovary (pathologic Stage I). Recurrence was diagnosed by computed tomography (CT)-guided biopsy of the paraspinal lesion; neither peritoneal nor lymph node involvement was detected. Metastasis to parenchymal organs is unusual for low-grade serous ovarian tumor, even after transformation to LGSC. In particular, to the best of our knowledge bone metastasis after a long disease-free interval in a patient who originally had pStage I ovarian SBT/APSTs has never been reported. We checked tissue samples from both metastatic and primary sites for somatic KRAS and BRAF mutations to facilitate prediction of the prognosis and response to chemotherapy of our patient.

## Case presentation

### Clinical course

**Fig. 1:**
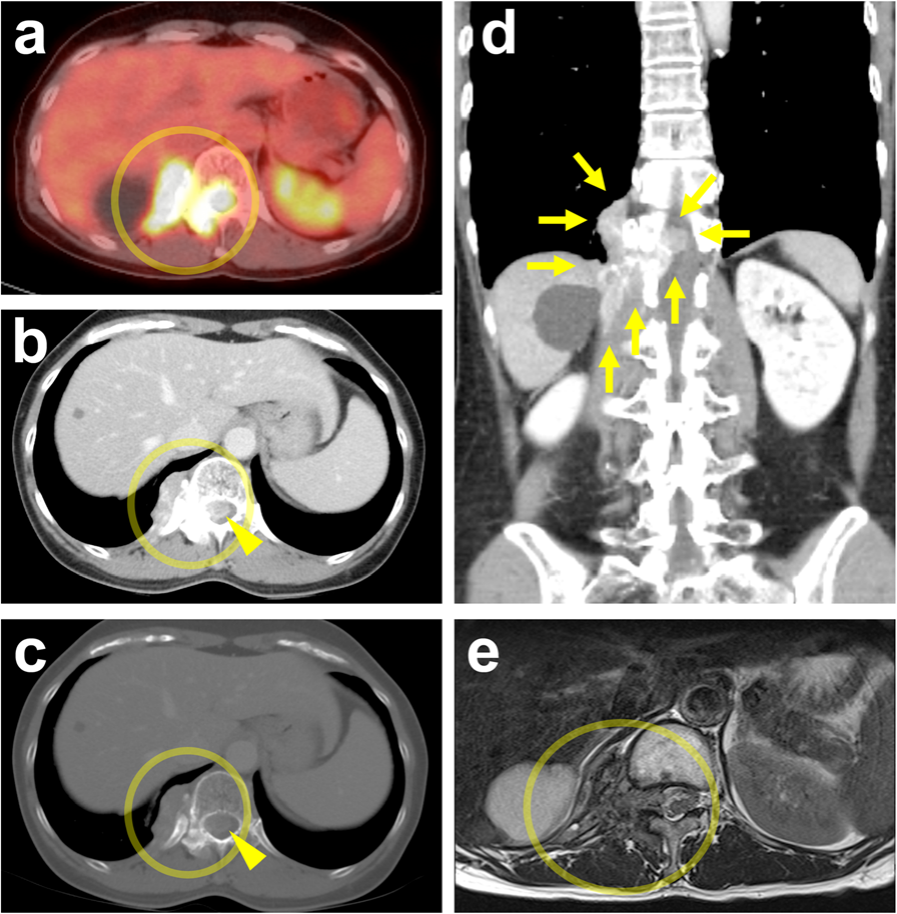
Imaging of the paraspinal lesion. **a** Positron emission tomography-CT fusion image demonstrating high fluorodeoxyglucose uptake (SUVmax 6.6). **b** and **c** Axial CT image showing a mixed osteosclerotic and osteolytic mass in the right arch of the Th12 vertebra (circled) on soft tissue (**b**) and bone (**c**) windows, protruding into the spinal canal (arrow heads). **d** Coronal reconstruction CT image revealing a bone metastasis infiltrating into surrounding soft tissue with peritoneal penetration through the diaphragm. **e** T2-weighted MRI image showing slightly high signal intensity in the same location (circled)

**Fig. 2:**
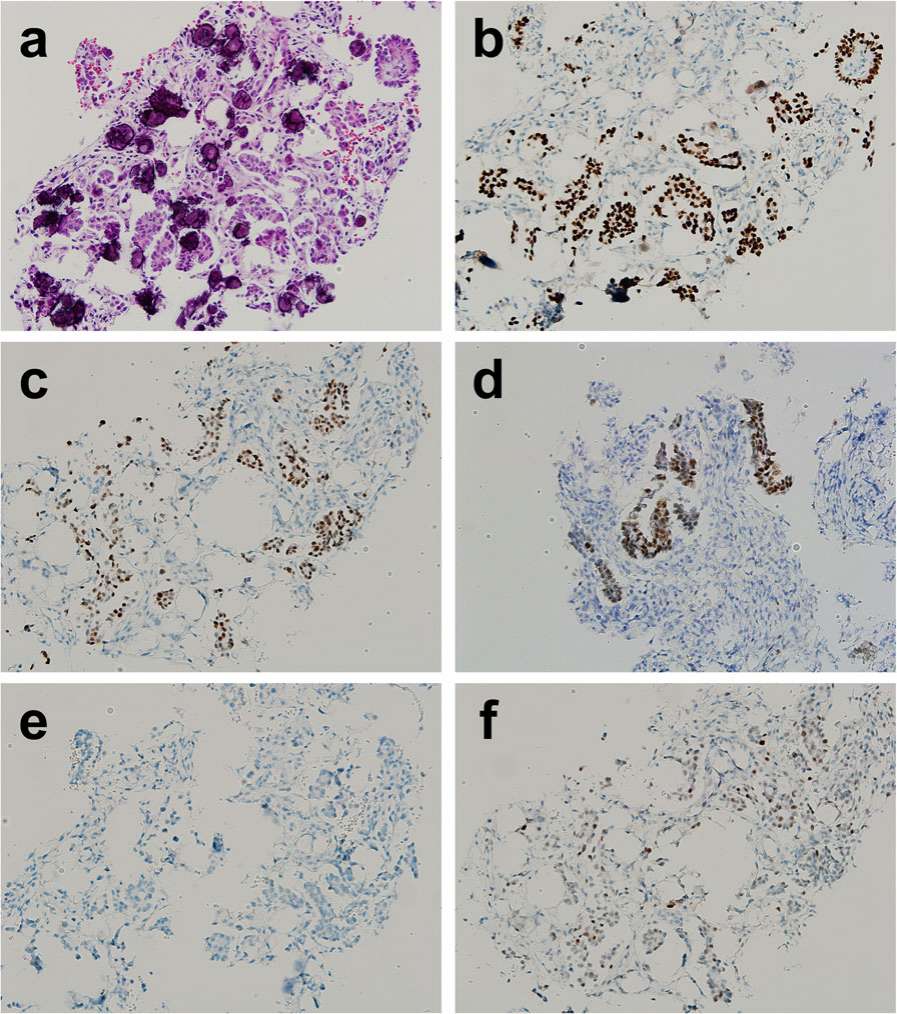
Photomicrographs of CT-guided needle biopsy of paraspinal neoplasm. **a** Small uniform columnar or round cells can be seen forming confluent papillae associated with psammoma bodies against a fibrous background (hematoxylin and eosin [HE] stain, × 200). **b** Neoplastic cells positive for PAX8 (immunohistochemical [IHC] stain, × 200). **c** Positive for ER (IHC, × 200). **d** Positive for WT1 (IHC, × 200). **e** Negative for thyroglobulin (IHC, × 200). **f** P53 wild-type expression (IHC, × 200)

**Fig. 3:**
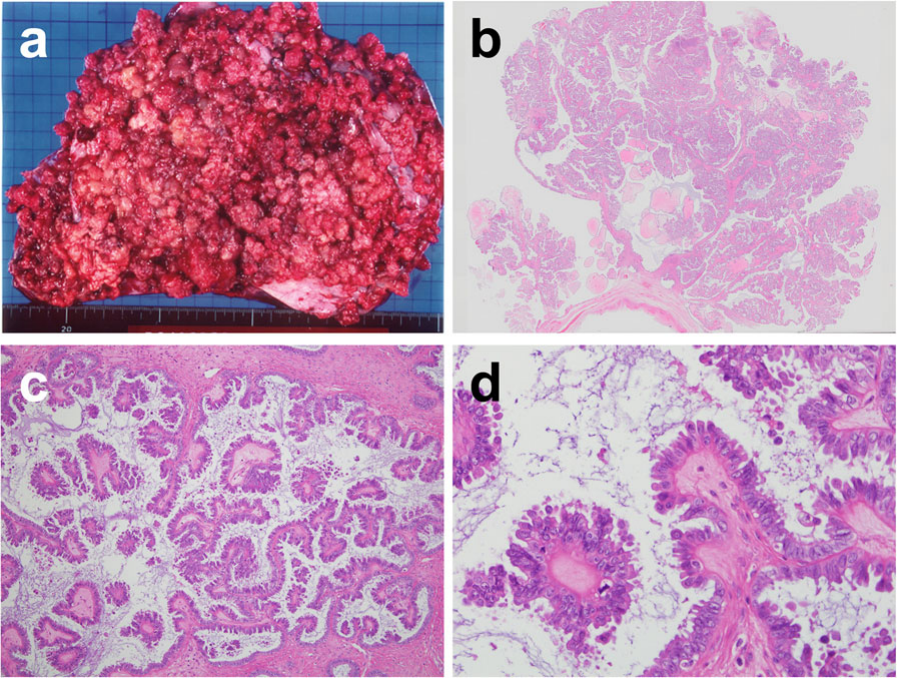
Original tumor in the right ovary **a** Photograph of operative specimen showing a soft tan, friable, intracystic and exophytic papillary tumor with occasional confluent excrescences. **b** Photograph showing of operative specimen showing delicate papillae with irregular contours but no detectable surface involvement or stromal invasion (HE, loupe view). **c** Photomicrograph showing hierarchical architecture with larger papillae branching into smaller ones with detached cell clusters or single cells (HE, × 100). **d** Photomicrograph showing ciliated and secretory cells with scanty cytoplasm and oval to round nuclei with finely distributed chromatin and rare mitotic figures. (HE, × 400)

A 55-year-old Japanese woman had developed right-lateral chest and back pain 2 months prior to admission to our hospital. Thirteen years previously, she had undergone concurrent bilateral salpingo-oophorectomy and total hysterectomy for right ovarian tumor in other institution. She had no past history of other neoplasms. Physical examination revealed deep-tendon hyperreflexia in the lower extremities but no muscle weakness. Blood tests were unremarkable apart from a high serum concentration of cancer antigen 125 (134 U/mL; cutoff value, 35 U/mL). On fluorodeoxyglucose (FDG) positron emission tomography the maximum standardized uptake value (SUVmax) was 6.6 in the right paraspinal region at the level of Th12 (Fig. 1a) and 8.8 in the right thyroid lobe; however, fine-needle aspiration cytology of the latter yielded no evidence of malignancy. CT scan and magnetic resonance imaging (MRI) revealed an irregular mass in the right arch of Th12 vertebral bone that protruded into the spinal canal through the intervertebral foramen and was infiltrating surrounding soft tissue, the whole mass being of 35 × 78 × 36 mm (Fig. 1b-e). No primary tumor was detected in other organs or the abdominal or pleural cavities. Pathological examination of a CT-guided needle biopsy of the paraspinal lesion demonstrated papillary proliferation of epithelial cells with blunt nuclear atypia against a fibrotic background with psammomatous calcification (Fig. 2a). Immunohistochemically, the neoplastic cells were positive for paired box 8 (PAX8) (Roche Diagnostics; Basel, Switzerland), estrogen receptor (ER) (Roche Diagnostics), and Wilms’ tumor 1 (WT1) (Roche Diagnostics), but negative for thyroglobulin (Nichirei; Tokyo, Japan) (Fig. 2b-e), thyroid transcription factor 1 (TTF1) (Roche Diagnostics), progesterone receptor (Roche Diagnostics), S100 protein (Roche Diagnostics), and calretinin (Roche Diagnostics). Staining for P53 (Roche Diagnostics) showed a non-mutational “wild type” pattern (Fig. 2f). Because the lesion was suspected of being a metastatic neoplasm from the previous ovarian tumor, we obtained the relevant clinicopathologic and operative records and found that our patient had had a 13- × 11- × 9-cm cystic tumor that did not involve the ovarian surface (Fig. 3a). Peritoneal cytology was negative; however, the tumor had been ruptured intraoperatively. Lymph-node biopsy was not performed during the original surgery. The six slides available for microscopic review in retrospect, which included all the 6 sections (1 on each slide) submitted for the previous diagnosis of the primary tumor, showed cuboidal to columnar epithelium with minimal cytologic atypia proliferating in hierarchical branches with no invasive features, micropapillary components, or peritoneal implants (Fig. 3b–d), the diagnosis being conventional Stage IC1 SBT/APST according to the current World Health Organization (WHO) and International Federation of Gynecology and Obstetrics (FIGO) 2014 classifications. All the above-listed immunochemistry markers were identical in the previous ovarian tumor and the present paraspinal neoplasm, except for Ki-67 (Agilent Technologies; Santa Clara, CA, USA), which had labeling indices of 1 and 5%, respectively. It was therefore concluded that the paraspinal lesion was a bone metastasis of transformed LGSC originating from the previous SBT/APST in the right ovary. The patient underwent radiotherapy (30 Gray in 10 fractions) for the paraspinal lesion, which relieved her pain, followed by chemotherapy treatment with paclitaxel and carboplatin plus bevacizumab. She remains alive with right-sided pleural effusion and suspected metastases in the Th3 and Th4 vertebrae 168 months after the initial surgery.

### Investigation of mutations using formalin-fixed paraffin-embedded tissues

Neither the primary nor metastatic tumor harbored KRAS mutation (codon 12, 13, 59, 61, 117, or 146) according to polymerase chain reaction (PCR) using a reverse sequence-specific oligonucleotide methodology (LSI Medience; Tokyo, Japan), nor BRAF V600E mutation according to real-time PCR (LSI Medience).

## Discussion

The current WHO Classification of Tumors of Female Genital Organs 2014 introduced new terminology for two separate categories of ovarian serous carcinomas, namely low- and high-grade ones, the former being described as being on a continuous spectrum from cystadenoma to carcinoma via borderline tumor [1]. The important changes in classification of this entity include classifying SBT/APSTs with any invasive foci (previously known as “invasive implants”) as peritoneal LGSC and identifying a histologic variant as an SBT/APST with significant micropapillary features (size > 5 mm, currently synonymous with “non-invasive LGSC”) because its biological behavior more closely resembles that of ovarian LGSC than of conventional SBT/APST [2]. In a meta-analysis of 97 studies including 4129 patients with SBT/APST, Seidman et al. reported overall survivals of virtually 100% for Stage I tumors and 95.3% for advanced stage tumors with non-invasive implants, whereas the survival for tumors associated with invasive peritoneal disease, that is, LGSC, was only 66% after a 7.4-year follow-up [3]. A nationwide cohort study in Denmark of 1487 women with SBT/APST with or without micropapillary features over a 25-year period (1978–2002) found that overall survival of patients with tumors confined to the ovaries was not different from that of the general population [4]. Further review of the histopathological features of these patients’ tumors by a panel of pathologist resulted in classifying 867 of them as conventional SBT/APSTs and 75 as non-invasive LGSCs. In the entire cohort, 41 women (4%) developed invasive serous carcinoma (38 low grade, three high grade; median time to diagnosis, 10 years; range, up to 25 years), such progression occurring significantly more frequently in patients with non-invasive LGSCs than in those with conventional SBT/APSTs [5]. In the present case, we speculate that recurrence was attributable to microscopic residual tumor after debulking surgery, possibly related to seeding caused by intraoperative rupture, developing in an indolent manner during the 13-year disease-free period. Given that distant metastasis must have occurred via lymphovascular permeation and that no peritoneal foci were detected at the time of recurrence, we consider it more likely that there was unrecognized lymph node involvement because lymph-node biopsy was not done during the original surgery. An alternative explanation is undersampling of the primary tumor (6 sections in all per a 13-cm tumor), in that the absence of invasive foci or micropapillary components in the tissue sections examined histologically did exclude their presence in unsampled parts of the primary tumor. Though additional sampling may not have changed the overall diagnosis, it would have provided credence to the assertion that no invasion or micropapillary features were identified.

Immunohistochemistry is essential for histologic diagnosis of suspected metastases, especially with unknown primary tumors. In women with a history of gynecologic tumor and proliferative cells in a papillary arrangement in a metastasis, PAX8 is very helpful in that it is a highly specific marker for neoplasms originating in the Müllerian duct system [6], whereas strong WT1 positivity points toward extra-uterine serous differentiation [7]. In our patient, another possibility, thyroid cancer, was excluded by the finding of negativity for both thyroglobulin and TTF1, the former being highly specific to follicular neoplasms with the exception of poorly differentiated and anaplastic types [8]. The radiologically-determined location of the tumor was consistent with aggressive meningioma; however, this possibility was ruled out because the morphology was not typical of either conventional or papillary subtypes and both of these are usually negative for PAX8 and WT1. Negative calretinin staining ruled out mesothelioma. The presence of wild-type expression of P53 excluded high-grade serous carcinoma (HGSC) [9]. Thus, we made a final diagnosis of metastatic LGSC transformed from SBT/APST originating in the right ovary.

To the best of our knowledge, no patient with initially diagnosed SBT/APST confined to the ovary and subsequently presenting with a lesion in bone as the first metastatic site has previously been reported. Intraparenchymal metastasis, that is, neither peritoneal nor lymph node involvement, is quite uncommon in early low-grade serous ovarian tumors, whereas metastases can occur in various extraperitoneal sites in advanced stage LGSCs [10–12]. Xing et al. recently reported a 59-year-old woman with a history of breast cancer who developed metastatic SBT/APST in the brain, this being considered to have originated in serous cystadenofibromas of both ovaries excised 5 years after a brain metastasis of unknown primary had been diagnosed [13]; thus, pathologists should take into consideration that SBT/APSTs lacking any LGSC features can metastasize to parenchymal organs in an occult fashion. Given that time to relapse after initial diagnosis of SBT/APST or LGSC has been reported to be as long as 28 years [14], long-term surveillance should be continued in women with SBT/APST and LGSC, which unpredictably manifest as overt metastases after a long disease-free period.

Somatic mutations in KRAS or BRAF occur separately or not all (never simultaneously) in about 50% of SBT/APSTs and LGSCs [15, 16], whereas P53 mutations occur almost exclusively in HGSCs [9]. Wong et al. reported detection of BRAF or KRAS mutations in nine (30%) and five (17%), respectively, of 30 SBT/APSTs, but in only one (2%) and eight (19%), respectively, of 43 LGSCs. BRAF mutations occurred less frequently at advanced stages [17]. The same group detected KRAS mutations with PCR and Sanger sequencing in 10 (43%) of 23 women with recurrent LGSCs (six in primary SBT/APSTs, five in recurrent LGSCs, and one in both), whereas only one patient had BRAF V600E mutation in a SBT/APST [18]. Because advanced stage and recurrent LGSCs are usually chemoresistant, clinical trials have been performed on new chemotherapeutic agents correlated to genetic profiles of individual tumors. LGSCs harboring BRAF or KRAS mutations often respond to mitogen-activated protein/extracellular signal-regulated kinase inhibitors such as selumetinib via down-regulation of the MAPK pathway. However, there is reportedly no correlation between BRAF/KRAS mutations and objective response [19]. Bevacizumab in conjunction with chemotherapy and hormonal therapy is one recommended combination for BRAF/KRAS wild-type SBT/APST or LGSCs, as in the present patient [20].

## Conclusions

In summary, we here present an unusual case of a woman with Stage I ovarian SBT/APST having transformed into LGSC, who presented with a symptomatic bone metastasis 13 years after optimal reduction surgery and whose primary tumor was found on pathologic review to have no features of LGSC. Pathologists and radiologists should not exclude remote recurrence of ovarian SBT/APST even when neither peritoneal nor intraparenchymal foci are apparent. In addition to adequate sampling when examining the histology of the primary tumor, follow-up for decades is recommended for patients with conventional SBT/APST of the ovary.

## Abbreviations

CT: Computed tomography
ER: Estrogen receptor
FDG: Fluorodeoxyglucose
FIGO: International Federation of Gynecology and Obstetrics
HE: Hematoxylin and eosin
HGSC: High-grade serous carcinoma
IHC: Immunohistochemistry
LGSC: Low-grade serous carcinoma
MRI: Magnetic resonance imaging
PAX8: Paired box 8
PCR: Polymerase chain reaction
SBT/APST: Serous borderline tumor/atypical proliferative serous tumor
SUVmax: Maximum standardized uptake value
TTF1: Thyroid transcription factor 1
WHO: World Health Organization
WT1: Wilms’ tumor 1
